# Supplementary material for: Contamination of medical devices and hospital environments with free-living amoebae: Evidence from hospitals in Northwestern Iran
Source: PLoS One. 2026 Apr 21;21(4):e0344500. doi: 10.1371/journal.pone.0344500 (PMC13098908; doi:10.1371/journal.pone.0344500)
Supplement: S1 Table — (DOCX) [file pone.0344500.s001.docx]

S1 Table. Exact source of samples.

|  | Code | Source |
| --- | --- | --- |
|  |  |  |
| Ophthalmology hospital | 1 | Ventilator |
|  | 2 | Desk |
|  | 3 | Surface (wall) |
|  | 4 | Oven |
|  | 5 | Bed |
|  | 6 | Prob of laser |
|  | 7 | Bed |
|  | 8 | Phaco |
|  | 9 | Prob of laser |
|  | 10 | Microscope |
|  | 11 | Desk |
|  | 12 | Swab |
|  | 13 | Autoclave |
|  | 14 | Dish of surgical instruments |
|  | 15 | Rat tooth forceps |
|  | 16 | Bed |
|  | 17 | Surface (wall) |
|  | 18 | Vitrectomy probe |
|  | 19 | Surface (bottom) |
|  | 20 | Surface (bottom) |
|  | 21 | Nursing Station |
|  | 22 | Surface (wall) |
|  | 23 | Forceps |
|  | 24 | Scissors |
|  | 25 | Chair |
|  | 26 | Surface of refrigerator |
|  | 27 | Pillow of patients |
|  | 28 | Pillow of patients |
|  | 29 | Surface of refrigerator |
|  | 30 | Surface of refrigerator |
| General hospital | 30 | Urinary catheterization |
|  | 31 | Scalpels |
|  | 32 | Angiocatheters |
|  | 33 | Protective shields |
|  | 34 | Angiocatheters |
|  | 35 | Angiocatheters |
|  | 36 | Protective shields |
|  | 37 | Surgical gowns |
|  | 38 | Scissors |
|  | 39 | Protective shields |
|  | 40 | Forceps |
|  | 41 | Surgical gowns |
|  | 42 | Angiocatheters |
|  | 43 | Autoclave |
|  | 44 | Surgical gowns |
|  | 45 | Protective shields |
