# Supplementary material for: Contamination of medical devices and hospital environments with free-living amoebae: Evidence from hospitals in Northwestern Iran
Source: PLoS One. 2026 Apr 21;21(4):e0344500. doi: 10.1371/journal.pone.0344500 (PMC13098908; doi:10.1371/journal.pone.0344500)
Supplement: Attachment — Submitted filename: Responses to Reviewers.docx [file pone.0344500.s003.docx]

**Response to Editors (Journal Requirements)**

Dear Editors,

Thank you for considering our manuscript (PONE-D-25-43069) and for the opportunity to revise. We have addressed all requirements as follows (All mentioned line numbers correspond to your comments; therefore, they are consistent with the previous version.):

1. We have reformatted the manuscript using PLOS ONE's style templates, including proper file naming (e.g., Manuscript.docx, Figure1).
2. As this study involved only environmental and equipment samples with no human participants, medical records, or archived patient data, informed consent was not applicable. This was waived by the ethics committee (IR.SARAB.REC.1402.02). We have added this clarification to the Methods section and online submission.
3. The amended funding statement is: "The funders (Sarab Faculty of Medical Sciences, Grant No.: 401000013) had no role in study design, data collection and analysis, decision to publish, or preparation of the manuscript." Please update the online form accordingly.
4. We have included Table 4 (listing GenBank accessions) in the revised manuscript.
5. We evaluated suggested citations from reviewers and incorporated relevant ones (e.g., for methods and discrepancies) where they enhance the manuscript.

We believe these changes strengthen the manuscript and look forward to your feedback.

Kind regards

**Response to Reviewer #1**

Dear Reviewer #1,

Thank you for your detailed feedback. We have revised the manuscript extensively for language, clarity, organization, and scientific rigor. Point-by-point responses below:

- **Overall Language and Writing**: We conducted thorough editing, including professional language review, to eliminate garbled statements, improve formality, and ensure scientific tone. AI tools were used only for polishing, with all content authored by us. It is worth mentioning that we evaluated the current version using Grammarly, which yielded a score of 99 (the report is uploaded as other).
- **Scientific Names**: All scientific names are now italicized (e.g., *Acanthamoeba*).
- **Organization/Presentation**: Results, Discussion, and Conclusion restructured for better flow, depth, and implications (e.g., expanded on clinical risks).
- **Tables/Figures**: Improved quality; replaced low-quality images in Figure 1 with clearer ones and added arrows. Added captions. Constructed a single phylogenetic tree for all genera.
- **Line 45, 73**: Revised to "Acanthamoeba keratitis" (capitalized properly) throughout.
- **Line 59**: Italicized names.
- **Line 68**: Revised to "These amphizoic organisms exist as trophozoites or resistant cysts, enabling survival in harsh conditions."
- **Line 75**: Removed italics from genera when not binomials.
- **Line 77**: According to the dear reviewer #3's comment, the section "Introduction" was shortened, but the issue was clarified in the current version. “Instead of using traditional morphological names such as *Acanthamoeba polyphaga*, molecular characterization now enables the classification of *Acanthamoeba* isolates into genotypes (T1–T23).”
- **Line 88**: According to the dear reviewer #3's comment, the section "Introduction" was shortened, and this sentence was removed.
- **Lines 92-95**: In the new version of the “Introduction” reworded for coherence. “High-risk settings include ICUs and ophthalmology wards, where patients face surgical procedures amid potential contamination from water systems and equipment.”
- **Sample collection questions**:

**The question**: Why are there unequal samples coming from the two hospitals?

**Response:** We clarified that the ophthalmology hospital was oversampled (30/45) because it caters to many corneal cases and has a greater risk for Acanthamoeba exposure; this rationale and exact sampling dates are added to Methods.

**The question**: What is the basis for the number of samples?

**Response:** Sample size based on the expected 20-30% prevalence from prior studies; unequal due to focus on a high-risk ophthalmology hospital."

**The question**: What is the rationale for the collection period?

**Response:** Since all samples were collected from indoor environments, seasonal variations in temperature and humidity are unlikely to influence the occurrence of free-living amoebae. Therefore, the collection period was not selected based on seasonality but rather on logistical availability and accessibility of the sampled sites.

**The question**:

- **Abstract**:

**The question**: which were examined for the first time?

**Response:** The sentence has been revised to clearly distinguish between ready-to-use medical devices and equipment (e.g., lasers, swabs, forceps) and other hospital items such as beds and gowns. To our knowledge, this is the first study to examine FLAs in these medical devices and equipment.

**The question:** In the section on sample collection, there was no mention of surgical gowns; however, they were repeatedly stated in the abstract section. This is a major concern.

**Response**: We appreciate the reviewer's careful reading. We acknowledge that the word "gown" was mistakenly spelled as "gun" in the Materials and Methods section and Table 2, which resulted in the omission of surgical gowns from the sample collection description. This error has been corrected in the current revision to reflect that surgical gowns were included accurately, ensuring consistency between the abstract and the Materials and Methods section.

- **Line 139**: Added: "*E. coli* (ATCC 25922), 10^8 CFU/mL, 100 μL/plate."
- **Lines 140-142**: Revised: "After sealing with Parafilm, the plates were Incubated at room temperature for up to 8 weeks; examined daily from week 2 using a light microscope at 100x magnification for screening, and 400x for details".
- **Lines 145-146**: Clarified as " Additionally, the positive plates were subcultured for axenic cultures. To achieve this, a fungal-free agar block containing amoebae was cut and placed on a fresh medium to eliminate contamination."
- **Thermo- and Osmo-tolerance**: Rewritten with details.
- **Genomic DNA/PCR**:

**The comment**: What is the rationale for using the ITS1 and ITS2 primer set for Vahlkamphia? Then, in Table 1 (Heading and genus column), you identify Vahlkampfiidae and Naegleria for the ITS1 and 2 primers set. These two are different organisms.

**Response**: The Internal Transcribed Spacer (ITS) primer set was used because it effectively amplifies the DNA of members of the Vahlkampfiidae family, including Naegleria species, as previously reported in the study “Morphological and Molecular Survey of Naegleria spp. in Water Bodies Used for Recreational Purposes in Rasht City, Northern Iran.” In our study, the PCR assay targeted the ITS region using the forward primer 5′GAACCTGCGTAGGGATCATTT3′ and the reverse primer ITS2 5′TTTCTTTTCCTCCCCTTATTA3′.

To clarify this point and avoid confusion, we have replaced the genus name "Naegleria" with "Vahlkampfiids" in the revised version of the manuscript. This correction indicates that the primer set was applied for detecting Vahlkampfiidae family members rather than a specific genus.

- **The Comment:** Are you saying that you have three different primer sets, but only one thermal cycling condition for all three?

**Response:** Yes, three different primer sets were used in the study; however, since their annealing temperatures were very similar, we applied a single optimized PCR thermal cycling condition that was suitable for all three primer sets.

- **Lines 189-195**: Rewritten; added morphological details (e.g., acanthopodia for *Acanthamoeba*).
- **The comment:** 181-182 Now, here you mention Vahlkampfia sp instead of Naegleria. This is very poor writing and confuses the readers.

**Response:** As stated in the Results section, based on both microscopic and molecular analyses, the isolate was identified as Vahlkampfia sp., not Naegleria.

- **The comment:** You used two different organisms for your outgroup??? How can you use Naegleria as one of your outgroups when you mentioned it for detection in

**Response:** As our study included three different genera (Acanthamoeba, Hartmannella, and Vahlkampfia), two types of outgroups were used to enhance phylogenetic resolution and accuracy. *Hartmannella vermiformis* (syn. *Vermamoeba vermiformis*) sequences from our isolates served as one outgroup, while a Naegleria sp. sequence from GenBank was included as a routine reference outgroup commonly used in free-living amoebae phylogenetic analyses. Although the table one was revised in the current revision.

- **The comment:** 189-195 This section is poorly written and confusing. How can you be so sure of identifying genera already just by microscopic observation? What did observe? Cysts or trophozoites? What were the morphological features? Why mention mixed populations if you performed a subculture?

**Response:** Mixed contamination is a common occurrence in free-living amoebae (FLA) studies. The initial microscopic identification of genera was performed using Page’s taxonomic key (as referenced in the Materials and Methods section), which relies on characteristic morphological features of both trophozoites and cysts. We did not include the full description of Page’s key in the manuscript to avoid unnecessary length and repetition. To address mixed populations, all positive plates were subcultured to eliminate fungal contamination and to isolate a single genus per plate for subsequent molecular detection and confirmation.

- **The Comment**: Table 2 Vahlkampfia or Naegleria? Which is which? This error is recurring throughout the manuscript. This is a major error.

**Response:** In the previous version of the manuscript, the term Naegleria was inadvertently used in some sections, including Table 1, to represent members of the family Vahlkampfiidae. In the current revision, all such instances have been carefully corrected, and only Vahlkampfia or Vahlkampfiids are now used consistently throughout the text, tables, and figure legends to ensure taxonomic accuracy and clarity.

- **The comment:** Why is it that in certain instances, free-living amoebae (FLAs) such as Acanthamoeba, Vahlkampfia, and Hartmannella (Vermamoeba) were detected microscopically after culturing, yet the PCR results came back negative?

**Response:** This discrepancy may occur due to several reasons, including low organism load below the detection threshold of PCR, degradation of DNA during sample processing, presence of PCR inhibitors, or differences in viability and DNA integrity between cultured and original samples.

Studies comparing microscopic, culture, and molecular methods have shown that PCR assays are generally more sensitive, but negative results can still occur even when organisms are visualized microscopically. For example, Yera et al. (2007) reported that combined PCR assays for Acanthamoeba keratitis were more sensitive (94%) than microscopy (33%) or culture (7%), but discrepancies did occur due to technical factors and sample quality (Yera H et al., "Comparison of PCR, microscopic examination and culture for the early diagnosis and characterization of Acanthamoeba isolates from ocular infections," Eur J Clin Microbiol Infect Dis, 2007). Similarly, Lass-Flörl et al. (2013) found that microscopy-positive, PCR-negative specimens may arise due to fungal contamination, sample loss of viability, aggressive specimen processing, or analysis of samples from different areas (Lass-Flörl C et al., "Utility of PCR in Diagnosis of Invasive Fungal Infections," Mycopathologia, 2013).

These suggest that while PCR is a powerful tool, its sensitivity may be affected by sample quality, DNA extraction efficiency, and technical limitations, especially when compared to microscopic identification in cultivated FLAs.

- **The comment: Table 3 There is no mention in the manuscript of how osmo-thermo-tolerance is evaluated/rated. What do these gradings mean? This should be extensively explained in the methods section.**

Response: It was explained in the “Material and methods” section. The added sentence: “Growth was scored qualitatively as “‑” (no growth), “1+” (rare/sparse growth), “2+” (moderate growth), “3+” (robust growth) based on the area and trophozoite density after 7 days, following criteria adapted from prior studies.”

- **The comment: Results section Poorly written.**

**Response:** The result section was revised.

- **The comment: Osmo- and Thermo-tolerance test Provide image evidence for these assays.**

**Response:** Capturing and presenting images from osmo- and thermo-tolerance assays is not a routine practice in relevant parasitology studies, and such images generally do not provide additional information or increase the scientific value of the data. Standardized protocols for osmotolerance and thermotolerance assays in Acanthamoeba research primarily focus on growth outcomes and viability assessments, which are evaluated by observing and scoring the amoebae's ability to grow under defined osmotic or temperature stress, rather than relying on or publishing distinctive image evidence. Multiple peer-reviewed investigations have reported osmotolerance and thermotolerance results in tabular or descriptive formats, with endpoints defined by growth (+/-) rather than by photographic documentation. Therefore, as also concluded by Kahraman et al. (2024) and Hajialilo et al. (2016), providing image evidence is unnecessary and not a recognized methodological requirement for these assays in the field (1. Kahraman M, Akın Polat Z. Are Thermotolerant and Osmotolerant Characteristics of Acanthamoeba Species an Indicator of Pathogenicity?. Turkiye Parazitol Derg. 2024 Mar 5;48(1):15-20. doi: 10.4274/tpd.galenos.2024.92408. 2. Hajialilo E, Behnia M, Tarighi F, Niyyati M, Rezaeian M. Isolation and genotyping of Acanthamoeba strains (T4, T9, and T11) from amoebic keratitis patients in Iran. Parasitol Res. 2016 Aug;115(8):3147-51. doi: 10.1007/s00436-016-5072-8. Epub 2016 Apr 22. PMID: 27102637.)

- **The comment: 233-237 So what does this mean?**

**Response:** Comparing study results with previously published studies is routine.

- **The comment: Discussion Shallow and repetitive. Requires reorganization and overall improvement in depth and implications.**

**Response:** The discussion section was revised.

- **The comment: Conclusion Poorly written. Acknowledgments Poorly written.**
- **Response: Both of them were revised.**
- **The comment: Figures 1 B and C are of poor quality. Replace these photos. Place arrow marks on structures of interest.**

**Response:** The figure was improved

- **The comment**: The phylogenetic tree should not be restricted to Acanthamoeba only. Rather, a phylogenetic tree should have been constructed for all isolates.

**Response**: Thank you for your comment. The all identified isolates (*Acanthamoeba* sp T4, *Valhkampfia* and *Hartmannella*) were marked by asterisk (*) and included in the tree based on concatenated regions of 18S rDNA, ITS1, 5.8S rDNA.

- **The comment: Where are the GenBank accession numbers for the study isolates?**
- **Response:** The table was added.

Thank you for helping improve our work.

Sincerely yours

**Response to Reviewer #2**

Dear Reviewer #2,

Thank you for your constructive comments. We have addressed them as follows (All mentioned line numbers correspond to your comments; therefore, they are consistent with the previous version.):

- **L59**: Italicized *Acanthamoeba*.
- **L83**: Specified "T4 genotype."
- **L104**: Used "FLA" only after first mention.
- **L124**: Added "transported at room temperature."
- **L126-134**: Added supplementary table listing all samples.
- **L138**: Clarified: "Central 2 cm portion cut aseptically."
- **L139**: Italicized *Escherichia coli*.
- **L140**: Added: " They grow easily in conditions of low oxygen, like many other difficult conditions, and some strains even grow better in anaerobic conditions."
- **L141**: Specified "using light microscope."
- **L145**: Specified "fungal contamination."
- **L149**: Changed to "evaluate."
- **L150**: Clarified “cyst-saturated agar blocks”.
- **L151**: Changed to "°C."
- **L159**: Added: "By scraping and suspending in PBS."
- **L163**: Removed "to."
- **Table 1**: Corrected formatting; capitalized genera; but "JDP" primers, specifically JDP1 and JDP2, target the 18S rRNA gene of Acanthamoeba. "JDP" is not an acronym for a longer name but a designation widely recognized in the molecular detection of *Acanthamoeba*.
- **L181**: Specified "GenBank reference sequences for each genus."
- **The comment on L182:** **why not using blastn instead of the tree?**
- **Response**: A phylogenetic tree provides distinct advantages over BLASTn for species identification and evolutionary analysis. While BLASTn is excellent for quickly finding the closest sequence matches and initial identification, it fundamentally provides pairwise similarity and cannot reveal deeper evolutionary relationships or clarify species delimitation in the presence of closely related taxa or cryptic species. Phylogenetic tree-based methods allow for the resolution of relationships among multiple sequences, visualize lineage divergence, and identify monophyletic groups, which is crucial for accurate species discrimination, understanding evolutionary histories, and addressing database errors or misidentifications that BLASTn alone may miss. Scientific studies recommend tree-based approaches (such as Maximum Likelihood or Bayesian inference) to robustly discriminate closely related or novel species, whereas BLASTn may misassign sequences due to reliance on the closest hit, especially in cases of database errors or incomplete reference datasets.
- **L191**: Corrected to Table 2.
- **L202**: Described levels: " Notably, two isolates demonstrated sensitivity to osmolarity, whereas the remaining isolates exhibited varying degrees of resistance to both osmolarity and heat shock. Specifically, Thermo-tolerance ranged from + at 37 °C to 2+ at 41 °C, while Osmo-tolerance ranged from - at 0.5 M to 1+ at 1 M (Table 3)."
- **Table 2**: Yes, code = sample number; clarified in caption.

Thank you for your insights.

Sincerely yours

**Response to Reviewer #3**

Dear Reviewer #3,

Thank you for your valuable suggestions. Responses below (All mentioned line numbers correspond to your comments; therefore, they are consistent with the previous version.):

1. Introduction shortened by condensing details.
2. Added references for tests (e.g., Khan, 2006).
3. Updated reference to Pélandakis & Pernin (2002) for Vahlkampfiidae/Naegleria. It is important to note that the primer can detect both Vahlkampfiidae and Naegleria.
4. Corrected numbering; added missing Table 2 (now Table 4 for accessions).
5. According to the journal's guidelines, "Figure captions are inserted immediately after the first paragraph in which the figure is cited. Figure files are uploaded separately." Consequently, I have placed the caption for Figure 1 immediately following its citation in the manuscript and have uploaded the figure separately.
6. In accordance with the aforementioned guideline, I have initiated the caption for Fig. 2 on line 214 of the previous version; thus, the inclusion of "Fig. 2" is essential.
7. Used "*Vermamoeba vermiformis*" consistently; removed "Hartmannella."
8. Replaced with higher-quality images.
9. According to the guideline of the journal I have placed the caption for the phylogenetic tree (Fig. 2) immediately following its citation in the manuscript and have uploaded the figure separately.

Thank you for improving our manuscript.

Sincerely yours
