# Supplementary material for: Contamination of medical devices and hospital environments with free-living amoebae: Evidence from hospitals in Northwestern Iran
Source: PLoS One. 2026 Apr 21;21(4):e0344500. doi: 10.1371/journal.pone.0344500 (PMC13098908; doi:10.1371/journal.pone.0344500)
Supplement: Attachment — Submitted filename: PONE-D-25-43069_reviewe. December 29, 2025.docx [file pone.0344500.s004.docx]

Manuscript ID: PONE-D-25-43069

**Contamination of Medical Devices and Hospital Environments with Free-Living Amoebae: Evidence from Hospitals in Northwestern Iran**

The manuscript presents a valuable and relevant investigation into the presence of free-living amoebae (FLAs) in hospital environments, with a specific focus on an ophthalmology center and ready-to-use medical equipment. The study is well-structured, methods are appropriate, and the findings contribute significantly to the understanding of FLA contamination in healthcare settings, highlighting a potential infection risk. However, several points require clarification, correction, or minor additions to enhance the manuscript's clarity, accuracy, and impact.

**Abstract**

-Conclusion: It is recommended to briefly include the key finding regarding the resistance of most Acanthamoeba isolates to osmolarity and heat shock, as this relates to their potential pathogenicity and has implications for disinfection protocols.

-Results (Line 55-56): The sentence "The isolated microorganisms were identified as belonging to the genera Vermamoeba (9), Acanthamoeba (7), and Vahlkampfia (7)" should be clarified. Since samples exhibited mixed contamination, it should be specified that these numbers refer to "the number of positive samples in which each genus was identified" rather than the number of independent isolates.

**Introduction**

-Line 68: The phrase "survive very severely" is grammatically incorrect. Suggested revision: "survive under harsh conditions" or "are highly resilient."

-Lines 98-99: The sentence "With a high rate of cornea injury patients admitted to..." is unclear. It is suggested to rephrase for clarity: "**Given the high admission rate of patients with corneal injuries at the selected Ophthalmology Hospital (Tabriz Alavi Hospital), and considering that many are contact lens users or postoperative cases, the risk of AK is elevated, necessitating detailed research**."

-It would strengthen the introduction to briefly mention the known resistance (Resistance Mechanisms) of *Acanthamoeba* cysts to common disinfectants, which underscores the significance and practical relevance of the present study.

**Materials and Methods / Results**

-The quantitative criteria for reporting growth (e.g., 1+, 2+, 3+) in the osmo- and thermo-tolerance tests (Table 3) are not defined. A clear explanation of these semi-quantitative scales should be provided in the corresponding Methods section.

-Please ensure that all genus and species names are **italicized** consistently throughout the manuscript (e.g., *Vermamoeba*, *Acanthamoeba*, *Vahlkampfia*, *V. vermiformis*).

Species Name: The abbreviation "*H. vermiformis*"(???) appears in the text and in Figure 2's title. For consistency with the updated taxonomy used elsewhere in the manuscript, please use "V*. vermiformis*" throughout.

-Figures:

Figure 2 (Phylogenetic tree): The quality/resolution of the figure in the provided file is poor, making labels and bootstrap values difficult to read. Please provide a higher-resolution version for publication.

**Discussion**

The discussion on the discrepancy between microscopic and molecular results is appropriate. Consider expanding it slightly to include other potential factors, such as the different sensitivities of the methods (e.g., PCR can amplify DNA from non-viable organisms, while culture detects only viable ones, and vice versa).

Lines 264-270: When discussing effective disinfection agents (OCT, QACs) and autoclaving, it is useful to note that the efficacy of chemical disinfectants can be influenced by factors like concentration, contact time, and the presence of organic matter/biofilms.
