# Supplementary material for: Contamination of medical devices and hospital environments with free-living amoebae: Evidence from hospitals in Northwestern Iran
Source: PLoS One. 2026 Apr 21;21(4):e0344500. doi: 10.1371/journal.pone.0344500 (PMC13098908; doi:10.1371/journal.pone.0344500)
Supplement: Attachment — Submitted filename: Responses to Reviewers_R2.docx [file pone.0344500.s005.docx]

**Response to Reviewer #4**

Dear Reviewer #4,

We want to extend our sincere thanks for your thoughtful and encouraging review of our manuscript. Your positive assessment has been greatly appreciated, as it validates our efforts and motivates us to continue advancing research in this area. We have carefully addressed your specific suggestions in the point-by-point response below.

**Abstract**

**Comment:** Conclusion: It is recommended to briefly include the key finding regarding the resistance of most Acanthamoeba isolates to osmolarity and heat shock, as this relates to their potential pathogenicity and has implications for disinfection protocols.

**Answer:** It was included in lines 65 to 67 " In addition to the high prevalence of FLAs in the examined sources, most *Acanthamoeba* isolates were found to be resistant to osmotic stress and heat shock, which supports their pathogenic potential."

**Comment**: Results (Line 55-56): The sentence "The isolated microorganisms were identified as belonging to the genera Vermamoeba (9), Acanthamoeba (7), and Vahlkampfia (7)" should be clarified. Since samples exhibited mixed contamination, it should be specified that these numbers refer to "the number of positive samples in which each genus was identified" rather than the number of independent isolates.

**Answer**: Your mentioned part was revise as following (line 56 to 60). " Most of the examined sources (90%) had mixed contamination, including *Acanthamoeba*, *Vahlkampfia*, and *Veramoeba* (4), *Acanthamoeba* and *Vahlkampfia* (1), *Acanthamoeba* and *Veramoeba* (2), and *Veramoeba* and *Vahlkampfia* (2). Also, one source showed sole contamination with *Vahlkampfia*. "

**Introduction**

**Comment:** Line 68: The phrase "survive very severely" is grammatically incorrect. Suggested revision: "survive under harsh conditions" or "are highly resilient."

**Answer:** It was revised in accordance with the comment. Current version (line 74): "to survive under harsh conditions."

**Comment:** Lines 98-99: The sentence "With a high rate of cornea injury patients admitted to..." is unclear. It is suggested to rephrase for clarity: "Given the high admission rate of patients with corneal injuries at the selected Ophthalmology Hospital (Tabriz Alavi Hospital), and considering that many are contact lens users or postoperative cases, the risk of AK is elevated, necessitating detailed research."

**Answer:** Was revised according to your suggestion. Current version (lines 101 to 103): "Given the high admission rate of patients with corneal injuries at the selected Ophthalmology Hospital (Tabriz Alavi Hospital), and considering that many are contact lens users or postoperative cases, the risk of AK is elevated, necessitating detailed research."

**Comment:** It would strengthen the introduction to briefly mention the known resistance (Resistance Mechanisms) of Acanthamoeba cysts to common disinfectants, which underscores the significance and practical relevance of the present study.

**Answer:** Resistance Mechanisms of cysts were mentioned briefly. Lines 74 to 77: "*Acanthamoeba* cysts, as one of the most clinically important genera of FLAs, exhibit pronounced tolerance to many widely used disinfectants because their sturdy, double-layered cyst wall (comprising an outer protein–polysaccharide layer and an inner cellulose-enriched layer) substantially restricts penetration of biocidal agents".

**Materials and Methods / Results**

**Comment:** The quantitative criteria for reporting growth (e.g., 1+, 2+, 3+) in the osmo- and thermo-tolerance tests (Table 3) are not defined. A clear explanation of these semi-quantitative scales should be provided in the corresponding Methods section.

**Answer:** The criteria were explained. Lines 159 to 161. "Growth was scored qualitatively as "‑" (no growth), "1+" (rare/sparse growth), "2+" (moderate growth), "3+" (robust growth) based on the area and trophozoite density after 7 days, following criteria adapted from prior studies."

**Comment:** Please ensure that all genus and species names are italicized consistently throughout the manuscript (e.g., Vermamoeba, Acanthamoeba, Vahlkampfia, V. vermiformis).

**Answer:** In the current version, all scientific names are italicized.

**Comment:** Species Name: The abbreviation "H. vermiformis" (???) appears in the text and in Figure 2's title. For consistency with the updated taxonomy used elsewhere in the manuscript, please use "V. vermiformis" throughout.

**Answer:** It was corrected.

**Figures**

**Comment:** Figure 2 (Phylogenetic tree): The quality/resolution of the figure in the provided file is poor, making labels and bootstrap values difficult to read. Please provide a higher-resolution version for publication.

**Answer:** The new image was provided.

**Discussion**

**Comment:** The discussion on the discrepancy between microscopic and molecular results is appropriate. Consider expanding it slightly to include other potential factors, such as the different sensitivities of the methods (e.g., PCR can amplify DNA from non-viable organisms, while culture detects only viable ones, and vice versa).

**Answer:** The paragraph was extended. Line 309 to  315: "Intrinsic variation in assay performance may also play a role, because PCR identifies target nucleic acid sequences regardless of organism viability and can therefore amplify DNA derived from non‑viable cells that would not be detected by culture or other viability‑dependent methods. In contrast, culture‑based and similar techniques detect only viable organisms, so a low microbial burden, demanding growth requirements, or a viable‑but‑nonculturable state can lead to negative culture or microscopic results even when pathogen DNA is still detectable."

**Comment:** Lines 264-270: When discussing effective disinfection agents (OCT, QACs) and autoclaving, it is useful to note that the efficacy of chemical disinfectants can be influenced by factors like concentration, contact time, and the presence of organic matter/biofilms.

**Answer:** The paragraph was revised. Line 282 to 297: "A recent study indicates that octenidine dihydrochloride (OCT) and quaternary ammonium compounds (QACs) are two commercially available agents effective in eliminating *Acanthamoeba* trophozoites and cysts when applied for an appropriate contact duration (21). These substances are suitable for both skin and superficial applications in human, and their effectiveness is markedly affected by factors such as concentration (for example, cyst-specific EC50 values), duration of exposure, and the particular formulation used. In applied settings, chemical disinfectants may show diminished activity when contact time is insufficient, the active ingredient is below the optimal concentration, or organic material and biofilms are present, as these can shield microorganisms or neutralize biocidal agents. Although autoclaving at 121°C for 15 minutes is generally recommended as a standard sterilization procedure (28), experimental evidence indicates that this regimen may not invariably eliminate all *Acanthamoeba* cysts, highlighting the necessity of validating sterilization protocols for highly resistant protozoa. Accordingly, ongoing surveillance is required, since amoebae can be reintroduced and re-establish contamination on instruments between autoclaving cycles and subsequent use, particularly in environments prone to biofilm development and organic soiling on device surfaces."

Thank you for helping improve our work.

Sincerely yours

**Response to Reviewer #5**

Dear Reviewer #5,

Thank you very much for taking the time to review our manuscript and for your supportive remarks. We are particularly grateful for your positive evaluation, which reinforces the importance of our work and inspires further contributions to the field. Below, we provide a detailed point-by-point response to your comments.

**Comment:** Please carefully proofread the final (clean) manuscript to remove duplicated sentences and residual track-change artifacts, particularly in the Abstract and Introduction.

**Answer:** The manuscript was reviewed carefully, and all duplicated sentences and residual track-change artifacts were removed.

**Comment:** Consider simplifying or softening references to AI-assisted tools in the Acknowledgments section, as some editors may be sensitive to explicit mentions beyond language polishing.

**Answer:** The Acknowledgments section was revised according to this comment. Acknowledgments: "The authors sincerely thank the administrators, authorities, and staff of the Sarab Faculty of Medical Sciences and the two participating hospitals for their cooperation and assistance during sample collection. The authors also appreciate the use of language-support AI tools during manuscript preparation to improve clarity and readability. In addition, they acknowledge the statistical support provided by the Clinical Research Development Unit of Al-Zahra Hospital, Tabriz University of Medical Sciences.

**Comment:** Although acceptable for a cross-sectional exploratory study, the relatively small sample size should be clearly acknowledged as a limitation. Explicitly mentioning the absence of culture-independent methods (e.g., metagenomics) as a limitation would further strengthen the scientific transparency of the study.

**Answer:** A section titled "Limitations" was added, and all the limitations you mentioned were stated there. Limitation section: "The present study has some limitations that must be mentioned. The first limitation is the small sample size and limited sampling locations, which could affect the generalizability of the results; therefore, it is recommended that other similar studies be conducted. Furthermore, in this and similar studies, FLA detection relies solely on cultivation methods paired with specific molecular assessments applied to positive cultures, potentially leading to the loss of uncultivable or low-abundance amoebae. The absence of culture-free techniques, such as metagenomic analysis, represents a significant drawback, as these strategies could provide a more comprehensive and precise depiction of microbial populations in healthcare facilities. Despite these limitations, this study provides valuable information on the prevalence of FLA in Medical Devices and Hospital Environments."

Thank you for your insights.

Sincerely yours
