# Supplementary material for: Contamination of medical devices and hospital environments with free-living amoebae: Evidence from hospitals in Northwestern Iran
Source: PLoS One. 2026 Apr 21;21(4):e0344500. doi: 10.1371/journal.pone.0344500 (PMC13098908; doi:10.1371/journal.pone.0344500)
Supplement: Attachment — Submitted filename: Reviewer comments.pdf [file pone.0344500.s002.pdf]

Reviewer comments:

Contamination of Medical Devices and Hospital Environments with Free-Living Amoebae: Evidence from Hospitals in Northwestern Iran

General comments:

Overall, the manuscript requires strong language improvement.

There are several statements that do not make sense (garbled), are improperly stated, or could be stated in a more formal/scientific manner.

Language editing is strongly recommended.

Scientific names are not italicized.

Poor quality organization and presentation of results, discussion, and conclusion.

Suboptimal (low-quality) presentation of tables and figures.

Poor-quality writing pervades the entire manuscript, despite the assistance of AI tools.

It is evident that the manuscript was written in a rush and did not receive careful attention to details or the critical development of ideas/perspectives/implications from the study's findings.

Specific comments:

Line number<br>Page number	Specific comments
45, 73	acanthamoeba keratitis<br>REVISE TO: *Acanthamoeba* keratitis<br>Correct this throughout the manuscript.
59	Italicize scientific names throughout the manuscript.
68	The statement makes no sense.
75	Genera should not be italicized.
77	Naming or detecting? This applies not only to one genus of FLA but to all FLAs.
88	Improper statement.
92-95	Garbled statements.
Sample collection	Why are there unequal samples coming from the two hospitals?<br>What is the basis for the number of samples?<br>What is the rationale for the collection period?
Sample collection	Abstract:<br>This study investigated the presence of FLAs in hospital environments<br>47 and ready-to-use medical devices, including beds and gowns, which were examined for the<br>first time.<br><br>Among the positive samples, 5 were obtained from environmental sources, 4 from equipment, and 1 from surgical gowns.

	In this study, FLAs were isolated from patients' beds and surgical gowns for the first time,<br><br>In the section on sample collection, there was no mention of surgical gowns; however, they were repeatedly stated in the abstract section. This is a major concern.
139	What is the volume of *E. coli* suspension applied per plate?<br>What is the inoculum density of the *E. coli* suspension used?<br>What is the source and reference strain of the *E. coli*?
140-142	The statement makes no sense.<br>8 weeks?<br>What lens are you talking about? 100 X magnification? This is not possible to examine cysts and trophozoites.
145-146	You are simply referring to a subculture to obtain a homogeneous population.
Thermo- and osmo-tolerance tests	This section is poorly written.
Genomic DNA extraction and PCR amplification	This section is poorly written.<br>What is the rationale for using the ITS1 and ITS2 primer set for Vahlkamphia? Then, in Table 1 (Heading and genus column), you identify Vahlkampfiidae and Naegleria for the ITS1 and 2 primers set. These two are different organisms.<br><br>Vahlkampfiids should not be italicized.<br><br>Are you saying that you have three different primer sets, but only one thermal cycling condition for all three?
181-182	Now, here you mention Vahlkampfia sp instead of Naegleria.<br>This is very poor writing and confuses the readers.
186-187	You used two different organisms for your outgroup???<br>How can you use Naegleria as one of your outgroups when you mentioned it for detection in Table 1?<br>How can you use Vermamoeba as an outgroup when it is included in the FLA for detection in Table 1?<br>This is very poor writing.
189-195	This section is poorly written and confusing.<br>How can you be so sure of identifying genera already just by microscopic observation? What did you observe? Cysts or trophozoites? What were the morphological features?<br>Why mention mixed populations if you performed a subculture?

Table 2	Vahlkampfia or Naegleria? Which is which? This error is recurring throughout the manuscript. This is a major error.<br>Code 1: Why was Vahlkampfia negative for the molecular test<br>Code: Why was Vermamoeba negative for the molecular test?<br>Code 24: Why is this negative for the molecular tests?<br>Code 29: Why is Acanthamoeba negative for the molecular test?<br>Code 31: Why is Acanthamoeba negative for the molecular test?<br>Code 32: Why is Vahlkampfia negative for the molecular test?
Table 3	There is no mention in the manuscript of how osmo-thermo-tolerance is evaluated/rated. What do these gradings mean? This should be extensively explained in the methods section.
Results section	Poorly written.
Osmo- and Thermo-tolerance test	Provide image evidence for these assays.
233-237	So what does this mean?
Discussion	Shallow and repetitive.<br>Requires reorganization and overall improvement in depth and implications.
Conclusion	Poorly written.
Acknowledgments	Poorly written.
Figure 1	Figures 1 B and C are of poor quality. Replace these photos.<br>Place arrow marks on structures of interest.
Phylogenetic tree.	The phylogenetic tree should not be restricted to Acanthamoeba only.<br>Rather, a phylogenetic tree should have been constructed for all isolates.
GenBank accession	Where are the GenBank accession numbers for the study isolates?
	NOTHING FOLLOWS.
